# Physiological Dysregulation and Aging: Implications for Health Equity

**DOI:** 10.1093/geroni/igaa057.3169

**Published:** 2020-12-16

**Authors:** Sarah Forrester, Catarina Kiefe, Roland Thorpe

**Affiliations:** 1 University of Massachusetts Medical School, Worcester, Massachusetts, United States; 2 Johns Hopkins University, Baltimore, Maryland, United States

## Abstract

Social determinants of health (SDOH) are a major public health issue that affect the magnitude and prognosis of many diseases in the U.S., including cardiovascular disease and cognitive disorders of aging. These associations are especially deleterious among minority persons in the US due to consistently unfavorable social determinants in this population including socioeconomic position, residential segregation, and quality of education. Physiological dysregulation measures the “true global state” of an individual that includes, but goes beyond, chronological age, reflects the health burden produced, in part, by SDOH, and is associated with the development and prognosis of disease. A better understanding of how and when physiological dysregulation occurs may allow us to prevent or reverse dysregulation among the most vulnerable aging populations leading to improved health equity in cardiovascular and cognitive outcomes.

